# Transient topographical disorientation due to right‐sided hippocampal hemorrhage

**DOI:** 10.1002/brb3.1078

**Published:** 2018-08-23

**Authors:** Stephanie Irving, Cauchy Pradhan, Marianne Dieterich, Thomas Brandt, Andreas Zwergal, Florian Schöberl

**Affiliations:** ^1^ German Center for Vertigo and Balance Disorders DSGZ Ludwig‐Maximilians‐University Munich Germany; ^2^ Graduate School of Systemic Neuroscience (GSN) Ludwig‐Maximilians‐University Munich Germany; ^3^ Department of Neurology Ludwig‐Maximilians‐University Munich Germany; ^4^ Munich Cluster of Systems Neurology SyNergy Munich Germany; ^5^ Clinical Neurosciences Ludwig‐Maximilians‐University Munich Germany

**Keywords:** hemorrhage, hippocampus, navigation, stroke, topographical disorientation

## Abstract

**Introduction:**

Topographical disorientation is defined as the inability to recognize familiar or unfamiliar environments. While its slowly progressive development is a common feature of neurodegenerative processes like Alzheimer's dementia, acute presentations are less frequent and mostly caused by strategic lesions within the cerebral navigation network. Depending on the lesion site, topographical disorientation can originate from deficits in landmark recognition and utilization for route planning (egocentric navigation deficit), or disturbance of an overarching cognitive map of the spatial environment (allocentric navigation deficit). However, objective measurements of spatial navigation performance over time are largely missing in patients with topographical disorientation.

**Methods:**

We here report a 55‐year‐old patient with acute topographical disorientation as the single symptom of right‐sided hippocampal hemorrhage and present quantitative gaze‐monitoring head camera‐based analyses of his path‐finding strategy and visual exploration behavior in a real space navigation paradigm.

**Results:**

The patient exhibited severe allocentric and also egocentric navigation deficits during the acute phase, shown by higher error rates at finding target items. In addition, he showed a more extensive use of search saccades toward, and fixations on, landmarks that could potentially serve as spatial cues. These deficits had been completely compensated for after four months, when the patient performed unremarkably in the real space navigation task, and used even more strongly allocentric path optimization strategies than age‐matched controls.

**Conclusions:**

This case report highlights the integral function and right‐sided dominance of the hippocampal formation in the cerebral navigation network in humans. It shows that the cognitive map can be restored completely despite a residual hippocampal lesion, which illustrates the enormous plasticity of the cerebral navigation network in humans.

## 1 INTRODUCTION

Previous studies show self‐reported deficits of navigation abilities in about one‐third of patients with mild stroke. However, isolated topographical disorientation is an uncommonly reported chief complaint of acute cerebral lesions characterized by sudden deficits in the recognition of familiar surroundings (Aguirre & D′Esposito, 1999; Van der Ham, Kant, Postma, & Visser‐Meily, 2013). Lesions to various brain regions including critical hubs of the cerebral navigation network may result in this impairment (Claessen & van der Ham, 2017; Ekstrom, Arnold, & Iaria, 2014). Most often the right hippocampus or parahippocampus is involved. It has been proposed that topographical disorientation can originate from deficits in allocentric and/or egocentric spatial strategies (Claessen & van der Ham, 2017). The cognitive map theory posits that the right hippocampus mainly supports allocentric processing of space and is thus activated in more complex navigational situations (Burgess, Maguire, & O'Keefe, 2002; O'Keefe & Nadel, 1978). In contrast, egocentric navigation mainly relies on sequential distance and direction computations by means of landmark recognition and utilization, processed particularly in the parahippocampal and retrosplenial cortex (Epstein & Vass, 2013). In this case study, we report a spatially disoriented patient with an acute focal right‐sided hippocampal/parahippocampal hemorrhage, and the long‐term time‐course of his deficits as documented by quantitative analyses of path‐finding strategy and gaze behavior in a real space environment. We hypothesized that (a) allo‐ and egocentric navigation abilities would be impaired in the acute stage due to the anatomic localization of the lesion and (b) navigation deficits would compensate over time by plasticity mechanisms within the cerebral navigation network.

**Figure 1:**
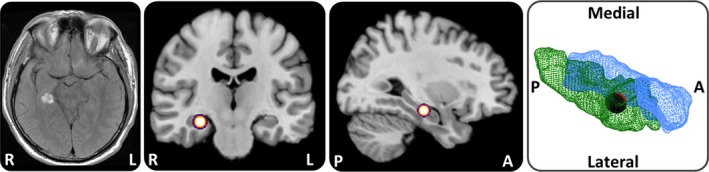
Lesion localization in a case of acute topographical disorientation. A T2 MRI sequence showed a focal hemorrhage in the right hippocampus and parahippocampus (left side). The lesion in full‐scale (red sphere) was projected to a standard T1 brain template (middle) and a hippocampus (blue) and parahippocampus (green) 3D model (right side) to visualize the exact lesion localization. R: right, L: left, A: anterior, P: posterior

**Figure 2:**
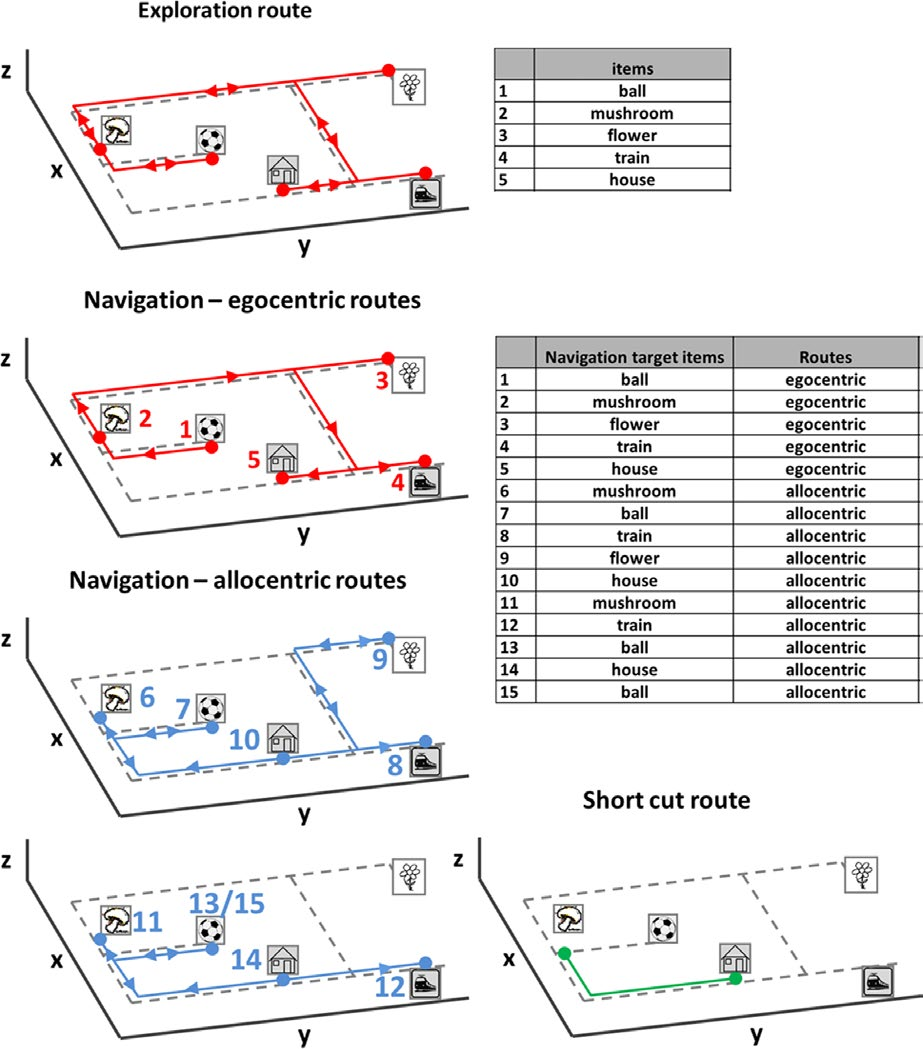
Navigation paradigm in real space. First row: exploration route with a defined sequence of items. Second row: In the first part of the navigation paradigm, routes were tested which were identical to the previous exploration route (so‐called egocentric routes). Third and fourth row: in the second part, the order of target items was changed in a way that required the planning of novel routes (so‐called allocentric routes). Potential short‐cuts within the allocentric route were recorded (fourth row, right side). The sequence of the target items during exploration and navigation is depicted in a table and appears as corresponding numbers besides the target items in the figures

The 55‐year‐old patient H.W. presented to the emergency room after feeling a sudden loss of familiarity with the entire surrounding environment while he was driving home from work. He reported no further subjective deficits, and in particular no amnesia, aphasia, apraxia or visual deficits. The neurological status was unremarkable except for a severe spatial orientation deficit. In particular, we could not find any signs of visual field deficits, neglect/extinction phenomena or deficits of left/right recognition. MRI revealed an acute focal hemorrhage, affecting the medial‐posterior hippocampus and adjacent parahippocampus (Figure 1). Detailed neuropsychological assessment was performed using the CERAD‐plus test battery. Performance in the subtests word list learning total, word list trial 1, 2 and 3, word list delayed recall, word list recognition, figure drawing, figure drawing recall and trail making test B was compared to age‐matched controls using *z*‐scores. H.W. furthermore underwent CLOX1 and CLOX2 tests to identify potential executive or visuospatial/visuoconstructive deficits. To exclude (hemi)neglect the Line Bisection and Balloons Test were performed. Results were depicted as deviation from the true center of lines for the Line Bisection Test and total B Score and Laterality B Index for the Balloons Test. Upon informed written consent by the patient and approval by the Ethics Committee of the Ludwig‐Maximilians‐University, Munich, topographical orientation was further assessed by an item search task in an unfamiliar real space environment. The environment, in which five items were placed as target points, was shown to the patient first by an investigator‐guided walk (exploration). Afterward, H.W. had to find the items in a pseudorandomized order within 10 min (navigation). The first part of the navigation paradigm was similar to the previous exploration route, which can be successfully solved by pure egocentric repetition of the route learned (i.e., sequential computations of distance and direction), thus requiring no cognitive map of the spatial environment. However, in the second part, the order of the target items was pseudorandomized, which consequently required detailed imagery of the environment as a whole and concrete planning of novel routes, potentially including short‐cuts (i.e., a cognitive map‐based or allocentric strategy) (Figure 2). Patient H.W. wore a gaze‐monitoring head camera throughout the experiment to document his visual exploration and head position (Schneider et al., 2009). To quantify spatial navigation performance, the error rate for items approached during the navigation phase was calculated by offline analysis of the videos recorded by the gaze‐monitoring head camera. Error rates were further separately analyzed for egocentric and allocentric routes to test for specific deficits of either navigation strategy. Error rates were compared to those of an age‐matched cohort of 10 healthy men (age: 54.3 ± 6.2 years). The search path during the navigation task was mapped by accumulating time spent at a specific place and analyzed quantitatively (mean gait speed during exploration or navigation, use of short‐cuts, and time spent at crossings). Video analysis allowed all fixation targets to be categorized into fixed objects in space, mobile objects, and unspecific, nonobject fixations (e.g., the ground, wall, ceiling). The total number of objects viewed and the number of unique objects viewed were also recorded. The objects, which were fixated most frequently, were plotted on a ground map to indicate the visual exploration strategy and landmark use. Analysis was carried out as described previously (Stuart et al., 2014; Zwergal et al., 2016). The total number of saccades and the saccade frequency were computed. Saccades directed to objects that were feasible as landmarks were defined as search saccades. The total number of fixations, fixation frequency, and duration were analyzed quantitatively. Fixation on a potential landmark was termed a search fixation. X and y gaze magnitudes and direction corresponding to the peak saccadic velocities and median fixation periods were identified and displayed as wind rose plots (direction and frequency of each class). All eye movement parameters were compared to data from the healthy controls.

Detailed neuropsychological assessment with the CERAD‐plus test battery indicated normal performance on all subtests. The respective *z*‐scores of subtest performance compared to age‐matched controls were as follows: word list learning total: 0.49; word list trial 1: 0.5, word list trial 2: 0.68; word list trial 3: −0.03; word list delayed recall: −0.09; word list recognition: 0.79; figure drawing: 0.7; figure drawing recall: −1.27; trail making test B: −0.76. The patient also showed a completely unremarkable performance on CLOX1/2 tests (15/15 points each). Neglect was ruled out by formally established tests such as the Line Bisection Test (deviation from true center of lines: 0.5 ± 1.1 mm) and Balloons Test (total B Score of 20 and Laterality B Index of 100%).

**Figure 3:**
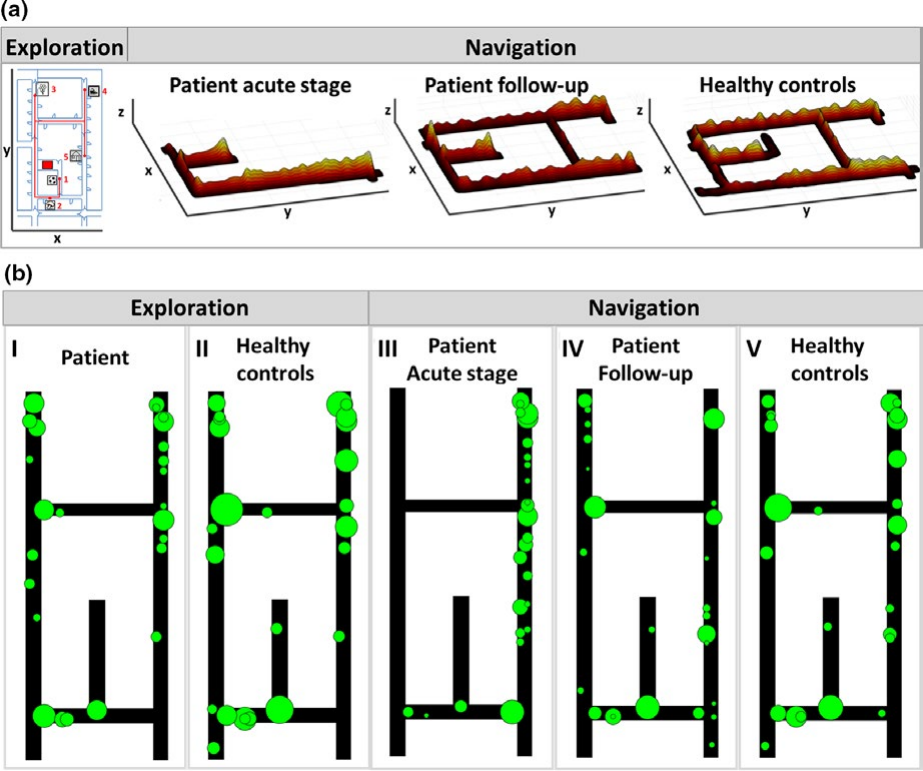
Navigational performance and visual exploration in the acute stage of topographical disorientation and during follow‐up after 4 months. (a) Navigograms of the patient were constructed by plotting the search path onto the floor map, with x and y indicating position in space and z accumulated time at place. The spatial position of the five search items (ball, mushroom, flower, train, house) is indicated on the left. During the acute stage of topographical disorientation the navigogram showed a severely impaired navigational strategy with complete loss of an internal cognitive map of the spatial environment. In the follow‐up examination 4 months later, the navigational performance was completely normal; the search path indicated an overall allocentric spatial strategy and was comparable to the group of healthy controls. (b) During guided exploration, the patient (I) and healthy controls (II) showed a similar pattern of object fixations. However, during navigation in the acute stage of topographical disorientation, the patient was not able to recognize these potential landmarks (III), whereas healthy controls showed a high consistency of retrieval of known objects (V). In follow‐up testing, the visual fixation pattern of H.W. normalized (IV) and got more similar to the strategy of healthy controls. Green circles indicate the most frequently fixated objects with position in space indicated on a ground map and diameters being relative to the total duration of fixation

**Figure 4:**
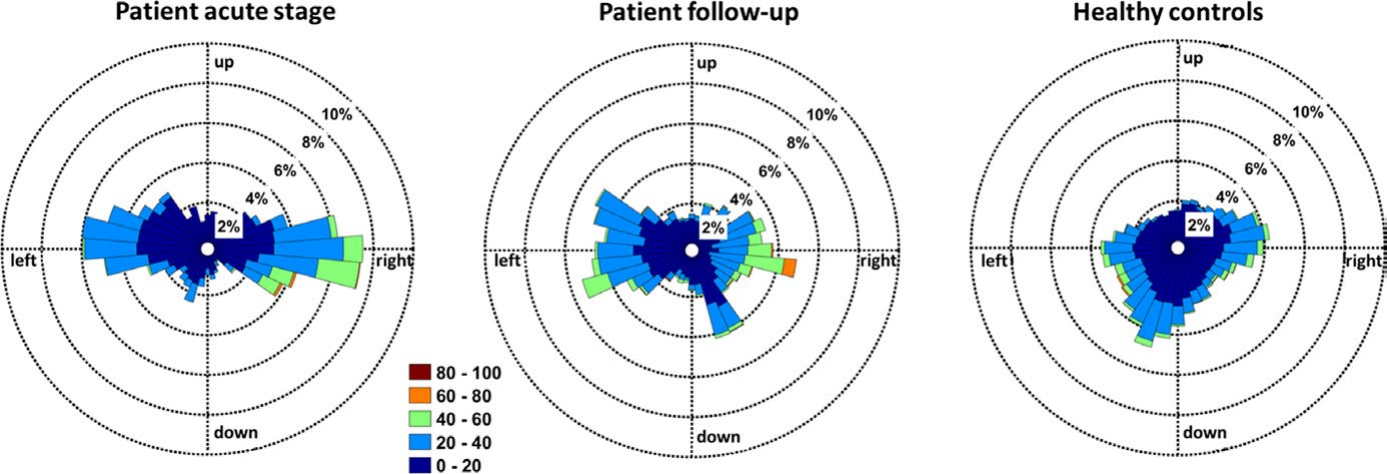
Visual exploration behavior in the acute stage of topographical disorientation and during follow‐up after 4 months and in healthy controls. Visual exploration behavior during navigation showed that saccadic eye movements during the acute stage of topographical disorientation were mainly directed to the lateral position (left panel) and there were more exploratory saccades. During follow‐up, saccadic eye movements were distributed more equally along the horizontal and vertical axes (middle panel). In healthy controls, saccades were directed mainly toward the ground and straight ahead (right panel)

On Day 2 after symptom onset, H.W.'s spatial navigation performance was severely impaired compared to that of an age‐matched control cohort: he had a higher error rate on both egocentric routes (patient: 100%, healthy controls: 5.0 ± 10.5%; *t*(9) = −28.500, *p* < 0.001) and allocentric routes (patient: 25%, healthy controls: 1.4 ± 4.5%; *t*(9) = −15.500, *p* < 0.001), stayed longer at crossroads (*t*(9) = 4.118, *p* = 0.003) and “neglected” half of the spatial environment (Figure 3a). Recognition of landmarks was impaired and fixations to objects were nonsystematic in the patient, while healthy controls had a high consistency in retrieval of strategically important landmarks (Figure 3b). The plot of H.W.'s visual fixations in space and the heat map of eye displacement from straight ahead position showed a pattern that was symmetric with respect to the lateral gaze position. This indicates that there was no visual neglect or asymmetric visual exploration behavior, but instead, that visual awareness was comparable across both visual hemifields (Supporting information Figure S1). The apparent asymmetry of navigation path trajectory likely resulted from the lack of any internal cognitive map of the surrounding environment and the distribution of visual cues/landmarks within it. However, given the restrictions of neuropsychological testing in the acute stage, the possibility of basic spatial working memory problems could not definitely be excluded. The patient chose the wrong turn at the beginning of the paradigm and from then just strayed up and down one hallway, unaware that there was another hallway parallel which he could explore. During navigation, he used more search saccades (*t*(9) = 3.613, *p* = 0.005) and tended to make more fixations to possible landmarks (*t*(9) = 2.085, *p* = 0.067). His gaze behavior was predominantly oriented within the horizontal plane (Figure 4, left). After an intense work‐up of possible bleeding etiologies, the patient was discharged and followed up four months later.

At that time, he reported no more problems with spatial orientation in his daily life. Therefore, H.W. performed the same real space navigation task like during the acute stage without any errors (egocentric routes *t*(9) = 1.500, *p* = 0.168 and allocentric routes *t*(9) = 1.000, *p* = 0.343), used even more short‐cuts than age‐matched controls (patient: 100%, healthy controls: 40.0 ± 46.07%; *t*(9) = −3.417, *p* = 0.008) and incorporated all possible paths into his navigational trajectory, thus highly indicative for the presence of an internal cognitive map of the spatial environment similar to healthy controls (Figure 3a). Landmark location and retrieval were similar to healthy controls (Figure 3b). Gaze behavior had also normalized, showing a more equal distribution of the vertical and horizontal planes (Figure 4, right). The numbers of search saccades (*t*(9) = 0.813, *p* = 0.437) and search fixations (*t*(9) = 1.179, *p* = 0.269) were comparable to that of age‐matched controls.

Combining behavioral measurements of navigational and neuropsychological performance, we here present evidence that a small, but strategic right‐sided hippocampal/parahippocampal lesion can cause acute topographical disorientation as a single symptom and can lead to severe deficits of both allo‐ and egocentric navigation strategies. This is remarkable as multiple brain areas form a distributed cerebral network for spatial navigation in humans (Ekstrom et al., 2014; Epstein & Vass, 2013; Grön, Wunderlich, Spitzer, Tomczak, & Riepe, 2000). This case underlines the integral function of the right hippocampus and parahippocampus within this network (Aguirre, Detre, Alsop, & D`Esposito, 1996; Byrne, Becker, & Burgess, 2007; Hartley, Maguire, Spiers, & Burgess, 2003; Morgan, Macevoy, Aguirre, & Epstein, 2011; Suthana, Ekstrom, Moshirvaziri, Knowlton, & Bookheimer, 2009). Therefore, hippocampal dysfunction in patient H.W. was indicated by a nearly complete loss of his internal cognitive map for space during the acute stage of symptoms (Hartley et al., 2003; Howard et al., 2014). An increase in search saccades together with a pronounced deficit in the recognition and incorporation of landmarks were associated with parahippocampal dysfunction (Aguirre et al., 1996; Epstein & Kanwisher, 1998). The pathological changes in gaze behavior and spatial navigation abilities in our patient resembled the pattern of other hippocampal navigation disorders such as MCI‐patients (unpublished data). Functional compensation of topographical disorientation in H.W. was rapid and complete, as reported in similar previous cases (Gil‐Néciga et al., 2002; Rivest, Svoboda, McCarthy, & Moscovitch, 2018). The complete recovery of an allocentric navigation strategy despite a residual hippocampal lesion in follow‐up MRI illustrates the great plasticity of the human navigation network (Byrne et al., 2007; Ekstrom et al., 2014). In the follow‐up assessment, the patient utilized an allocentric strategy with successful use of short‐cuts. Although it cannot be completely excluded that previous knowledge about the room representation alleviated the paradigm the second time, the most likely explanation may be a recruitment of extrahippocampal network structures as has been described earlier for hippocampal lesions (Kolarik, Baer, Shahlaie, Yonelinas, & Ekstrom, 2018; Maguire, Nannery, & Spiers, 2006).

In conclusion, acute topographical disorientation should be recognized in clinical practice as a distinct and focal symptom indicating right‐sided lesions of the hippocampal formation. The exceptional aspects of this case are the differentiation between egocentric and allocentric navigation strategies, the altered visual exploration behavior without any other signs for (hemi)neglect or any asymmetric visual exploration in general. Importantly, isolated and severe topographical disorientation due to a very strategic lesion as in our case can recover rapidly and completely without any sequelae. It can only be speculated how plasticity supported the recovery of topographical orientation—either through a functional substitution by the intact left hippocampus, or a fundamental reorganization within the broader human spatial navigation network including extrahippocampal hubs such as the retrosplenial and posterior parietal cortex.

## CONFLICT OF INTEREST

The authors declare that they have no conflict of interests.
