# Supplementary figures and images for: Transient topographical disorientation due to right‐sided hippocampal hemorrhage
Source: Brain Behav. 2018 Aug 23;8(9):e01078. doi: 10.1002/brb3.1078 (PMC6160660; doi:10.1002/brb3.1078)

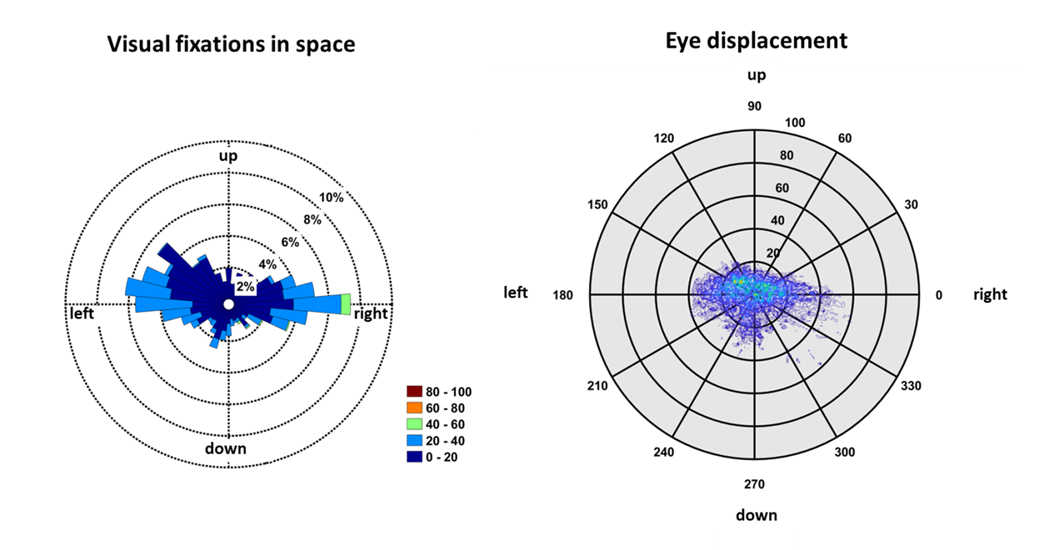

Supplement: Supplementary file 1 [file BRB3-8-e01078-s001.tif]
